# An anomalous abundance of tryptophan residues in ceramide synthases based on analysis of all membrane proteins in the Swiss-Prot database

**DOI:** 10.1016/j.jbc.2024.108053

**Published:** 2024-12-07

**Authors:** Beatriz Mestre, Iris D. Zelnik, Stav Izrailov, Tamir Dingjan, Gideon Lvovsky, Lena Fidel, Shifra Ben-Dor, Anthony H. Futerman

**Affiliations:** 1 Department of Biomolecular Sciences, Weizmann Institute of Science, Rehovot, Israel; 2 Department of Life Sciences Core Facilities, Weizmann Institute of Science, Rehovot, Israel

**Keywords:** sphingolipids, ceramide synthase, ceramide, lipid synthesis, tryptophan, membrane proteins

## Abstract

The relationship between membrane proteins and the lipid constituents of the membrane bilayer depends on finely-tuned atomic interactions, which itself depends on the precise distribution of amino acids within the 3D structure of the protein. In this regard, tryptophan (Trp), one of the least abundant amino acids, is found at higher levels in transmembrane proteins where it likely plays a role in helping anchor them to the membrane. We now re-evaluate Trp distribution in membrane proteins using all known proteins in the Swiss-Prot database and confirm that it is somewhat higher (∼1.7%) than in soluble proteins (∼1.0%), but not as high as in a well-quoted study (∼3.1%). However, the resident endoplasmic reticulum membrane protein, ceramide synthase (CerS), contains a higher abundance of Trp (3.4%). In the case of CerSs which contain a Hox-like domain, the Trp residues are asymmetrically distributed throughout the protein with a bias towards the lumenal side of the endoplasmic reticulum membrane. Mutation of these residues, even to other hydrophobic amino acids, leads to loss of activity, expression, and/or N-glycosylation. Moreover, five of the ten most conserved amino acids in the CerSs are Trp, and site-directed mutagenesis of numerous conserved Trp residues to alanine had distinct effects. Our data is consistent with other studies suggesting that Trp plays critical roles not only in membrane anchoring of transmembrane proteins but also in their activity and function.

The analysis of large datasets has become widespread in the biological sciences in the past decade or two, with the tools of artificial intelligence and machine learning now commonly used to interrogate such datasets (1). One of the most-widely used tools in protein science are those available *via* DeepMind’s AlphaFold server, such as AlphaFold2, which uses the sequences and structures of ∼100,000 known proteins to predict the 3D structure of a large number of proteins whose 3D structure has not been determined experimentally (2). To date, and despite the remarkable ability to predict protein tertiary structure, AlphaFold (and similar machine-learning based structure prediction methods) has not yet completely elucidated the interplay between sequence information, protein folding, and protein function.

One example of sequence information is the distribution of amino acids within protein sequences, with some amino acids much more abundant than others and others found at higher levels in certain regions of proteins (3). Thus, as might be expected, hydrophobic amino acids are more likely to be found in transmembrane domains (TMDs) embedded within and in contact with the membrane lipid bilayer (4, 5). By way of example, membrane proteins have been reported to have a significantly higher tryptophan (Trp) content than soluble proteins, and their distribution within the membrane is neither uniform nor random (6, 7). Some of the analyses on which this conclusion is based were taken from a study of only 20 membrane proteins and 72 cytoplasmic proteins from inner membrane proteins in bacteria nearly 40 years ago, and few if any studies (8) have re-evaluated this data in light of the much larger datasets available today.

In the current study, we analyze the distribution of all amino acids in > 210,000 nonredundant proteins available at Swiss-Prot (9). While previous studies indicated that Trp comprised 3.1% of the amino acids in membrane proteins (6), we now show that the actual percentage is considerably lower, at 1.7%. Unsurprisingly, the abundance of Trp in membrane proteins is higher than in soluble proteins, with no differences in percent abundance between proteins with one or with multiple TMDs. However, there are exceptions to this, with one being the ceramide synthases (CerSs). The CerSs are multi-spanning membrane proteins located in the endoplasmic reticulum (ER) which play a critical role in the pathway of sphingolipid biosynthesis by catalyzing the *N*-acylation of the sphingoid long-chain base (10, 11). Unexpectedly, the CerSs contain a significantly higher abundance of Trp residues than other proteins with a similar number of TMDs and a higher number than the Tram-Lag-CLN8 (TLC) family members to which the CerSs belong (12). We examined the distribution of Trp using AlphaFold2 (2) and also experimentally tested the effect of mutating many of the conserved Trp residues in the CerSs, revealing distinct effects on CerS activity and/or N-glycosylation. We conclude that the distribution of Trp residues in the CerSs is likely to play a key role in modulating CerS function and may also contribute to stabilizing the interaction of CerSs in the ER membrane due to the interfacial properties of Trp.

## Results

### Asymmetric distribution of Trp residues in CerSs

**Figure 1:**
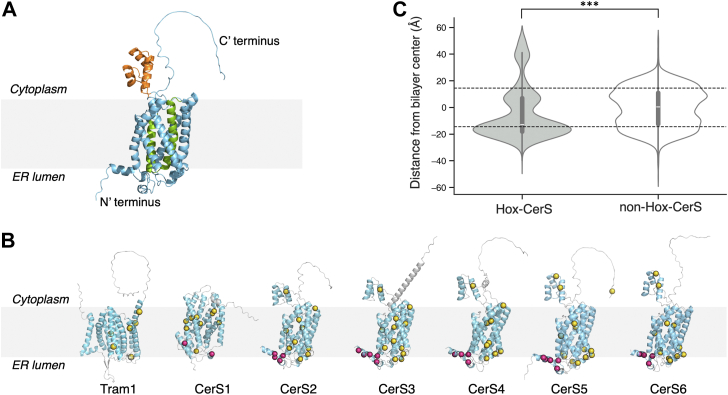
**Distribution and enrichment of Trp in the N-terminus of Hox-CerS.***A*, AlphaFold2 predicted structure of human CerS5 with the Hox-like (*orang*e) and Lag1p domains (*green*) indicated. The N′ terminus of the CerS faces the ER lumen while the C′ terminus faces the cytoplasmic side of the ER membrane. *B*, AlphaFold2 predicted structures of human Tram1 and CerS1-6. Trp residues in the N′ terminus are shown as *warm-pink* spheres while those in other regions of the protein are in *yellow*. Note that CerS1 lacks a Hox-like domain. *C*, violin plots of the distribution of Trp in Hox-CerS (*gray*; total of 66 sequences containing 1012 Trp residues) and in CerS that do not contain a Hox-like domain (*white*; total of 125 sequences containing 1365 Trp residues). Sequences were selected from the CerS clade [209 sequences from 84 species; Ref. (21)]. The vertical axis shows the distance of Trp Cα atoms (in Å) relative to the center of the bilayer (corresponding to 0 Å). The width of the violin plot depicts the distribution of Trp at different distances from the predicted bilayer center. The horizontal *white* bold line within each violin plot represents the median. The dashed *black* lines indicate the mean predicted boundary of the membrane lipid bilayer (at a distance of approximately 18 Å above and below the predicted bilayer center). Distances beyond +18 Å or −18 Å correspond to Trp residues predicted to be located in the cytoplasmic space and the ER lumen, respectively. Statistical comparison between the Hox-CerS and non-Hox-CerS was performed using the Mann–Whitney U rank test; ∗, *p* < 0.05; ∗∗, *p* < 0.01; ∗∗∗, *p* < 0.001.

The CerSs contain seven TMDs, with the N-terminus located within the lumen of the ER and the C-terminus located in the cytosol (Fig. 1*A*) (13, 14). The CerSs are characterized by two major structural domains, the TLC domain, a region of ∼200 residues (12) which contains a highly conserved stretch of 52 amino acids [the Lag1p motif (15, 16)], and a homeobox (Hox)-like domain, which is found in all mammalian CerSs with the exception of CerS1 (16, 17). Numerous loss-of-function mutations in CerSs have been described (17, 18), including two mutations of Trp residues [W15R (19) and W10M (20) in CerS3], which cause ichthyosis characterized by impairment of epidermal barrier function.

To explore the relationship between Trp mutations and the loss of CerS function, we examined the distribution of all Trp residues in human CerS using structural models obtained from AlphaFold2. An asymmetric distribution of Trp residues was observed in the five human CerSs containing a Hox-like domain (Hox-CerS), with more Trp residues located near the ER lumen, towards the N-terminus of the protein (Fig. 1*B*). In contrast, human CerS1, which does not contain a Hox-like domain, exhibited a nearly symmetrical Trp distribution, likely due to the lack of a Trp cluster in its N-terminus; similarly, Tram1 [a TLC family member (12)] exhibited a uniform distribution of Trp residues (Fig. 1*B*). Analysis of Trp distribution in a set of 191 CerS sequences [66 Hox-CerS and 125 non-Hox-CerS (21)] essentially confirmed the observation made in the human CerS inasmuch as two regions were observed with a higher abundance of Trp residues in Hox-CerS, namely the hydrophobic bilayer region of the ER membrane cytoplasmic leaflet (between +5 to +10 Å from the center of the bilayer) and the interface region at the ER lumenal leaflet (approximately −20 Å from the bilayer center) (Fig. 1*C*). In contrast, no such asymmetric distribution was observed in CerS lacking a Hox-like domain (Fig. 1*C*). The observed asymmetric distribution of Trp and the presence of a Trp-cluster at the N-terminus in Hox-CerS may reflect the ability of Trp to form hydrogen bonds, π-π stacking (22), π-cation (23), and π-anion (24, 25) interactions, which are crucial for stabilizing the membrane environment of the protein and contribute to the overall stability and function of membrane proteins (23).

### A conserved N-terminal Trp is essential for CerS activity and cannot be replaced by other aromatic amino acids

**Figure 2:**
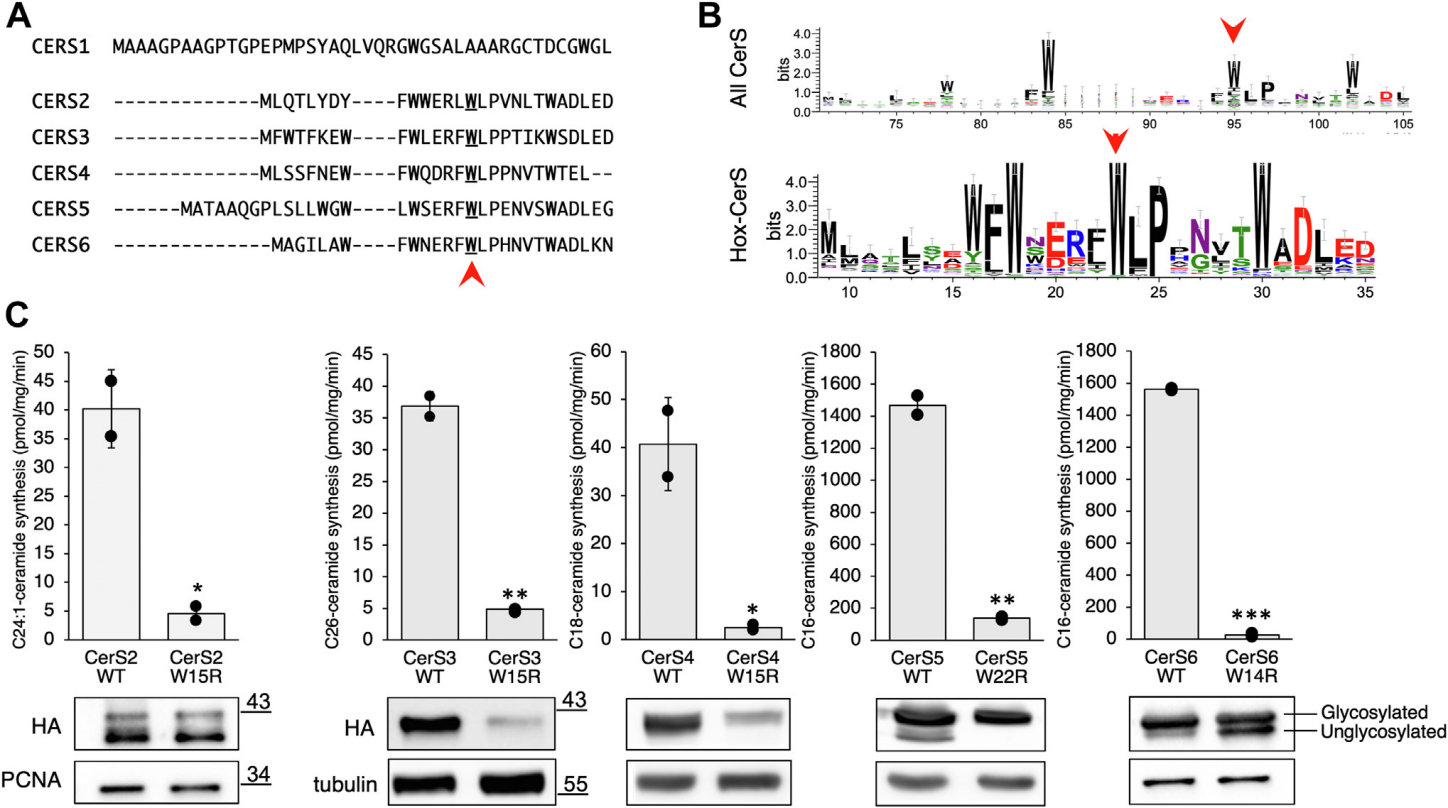
**Effect of mutating N-terminal Trp residues on CerS activity.***A*, sequence alignment of the N-terminus of human CerS1-6. The *red* arrow indicates the Trp residue of interest. Sequences were aligned using ClustalW. *B*, sequences of the N-terminus presented as a logo, which is a representation of the conservation of amino acids at each position. The height of the letter represents the dominance of that particular residue at that position; if there are no dominant residues, the position is blank or a combination of many small residues. The sequence logo was generated from 209 CerS1-6 homologous sequences from 84 species, ranging from amoeba to human, based on data from Ref. (21). When comparing alignments, the distances between residues in the logos vary due to the effect of the alignments from distant species. Arrows indicate the Trp of interest. The logo was built using WebLogo. *C*, homogenates were prepared from WT HEK cells overexpressing the indicated CerS, except for CerS2, which was overexpressed in HEK/CerS2^−/−^ cells. CerS activity was assayed using the indicated acyl-CoAs. CerS2 activity was assayed using 40 μg of protein from a cell homogenate for 25 min; CerS3 was assayed using 40 μg for 30 min; CerS4 was assayed using 35 μg for 25 min; CerS5 using 2 μg for 10 min; and CerS6 using 5 μg for 10 min. Results are means ± S.D. for a typical experiment repeated a minimum of three times in duplicate with similar results. ∗, *p* < 0.05; ∗∗, *p* < 0.01; ∗∗∗, *p* < 0.001. Levels of protein expression were ascertained by Western blotting using an anti-HA antibody. The glycosylated form is the upper band, and the nonglycosylated form is the lower band (14). Anti-tubulin (CerS3, 4, 5, and 6) or anti-PCNA (CerS2) were used as loading controls. Molecular weight markers (kDa) are indicated. Western blots were repeated at least three times with similar results.

The N-terminal region in human CerS2-6 is highly conserved (∼50–70% similarity and ∼40–50% identity) (11). CerS1 does not align with the other five CerS in this region as it belongs to a distinct branch of the phylogenetic tree, closer to the yeast CerS, Lag1, and Lac1 (26) (Fig. 2*A*). CerS2 and 4-6 are glycosylated at a conserved N-terminal motif (NXS/T), which is adjacent to the Trp cluster at the N-terminus. Although CerS3 lacks this conserved *N*-glycosylation motif and therefore is not modified by *N*-glycosylation, it also contains a Trp cluster at its N-terminus. We previously reported (19) that the W15R mutation in CerS3 resulted in a loss of enzyme activity [W15 is highly conserved across CerS proteins (Fig. 2*B*)]. We now mutated the equivalent Trp residues in CerS2, 4, 5, and 6, which resulted in loss of activity or expression in all cases (Fig. 2*C*). Upon overexpression of both CerS5^W22R^ and CerS6^W14R^ in HEK cells, different N-glycosylation patterns were observed, such that CerS5^W22R^ was only expressed as the glycosylated form while CerS6^W14R^ exhibited partial glycosylation. In contrast, CerS3^W15R^ and CerS4^W15R^ were expressed to a much lower extent than their WT counterparts, whereas levels of CerS2^W15R^ were similar to WT CerS2 (Fig. 2*C*). These results illustrate the functional significance of the conserved Trp residue on activity, expression, and glycosylation of CerSs, but the different impact that each mutation causes suggests more complex modes of regulation of CerS function than previously appreciated.

**Figure 3:**
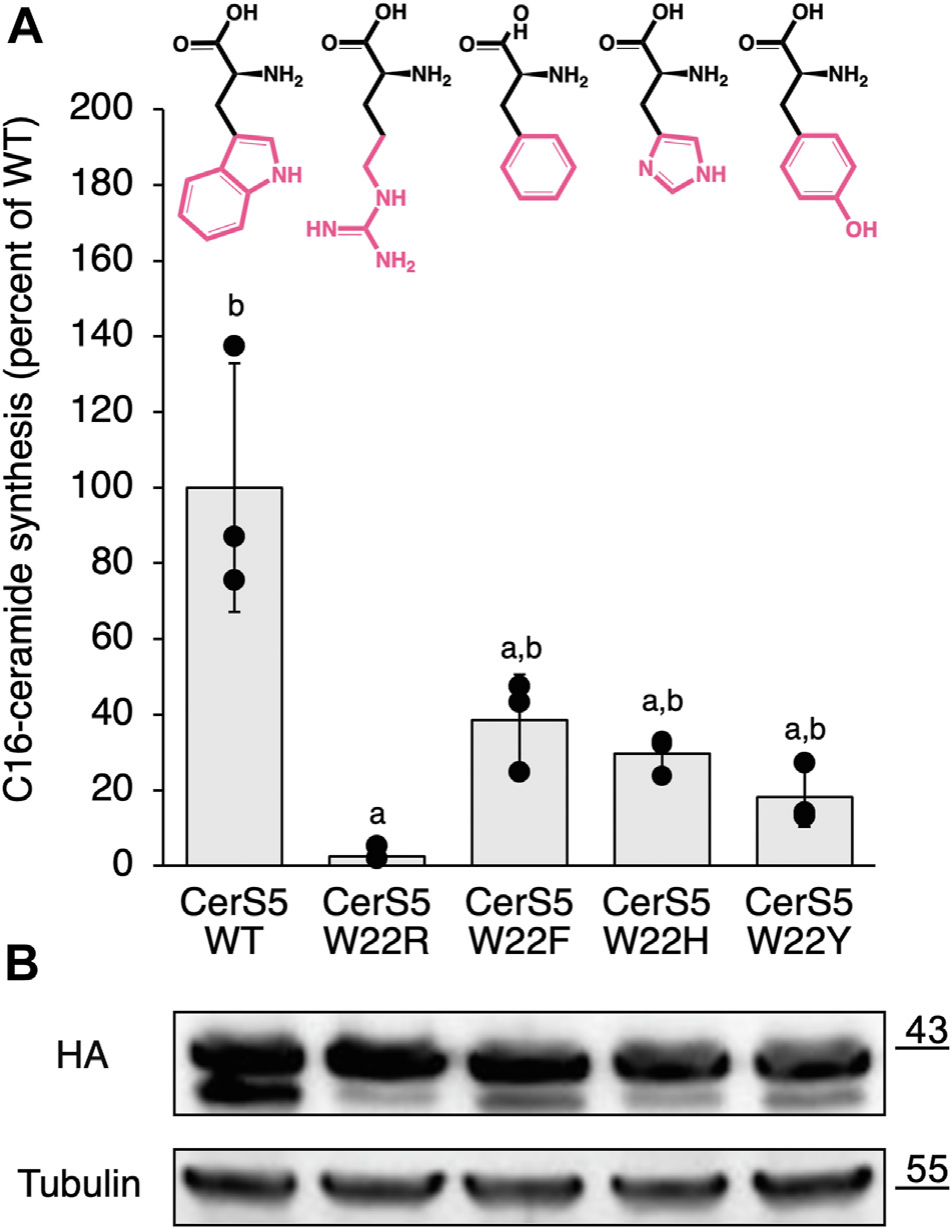
**The effect of mutating CerS5**^**W22**^**to other aromatic amino acids.***A*, homogenates were prepared from WT HEK cells overexpressing CerS5 with the indicated mutations. CerS activity was assayed using C16-acyl-CoA with 1 μg of protein from cell homogenates for 5 min. Results are percent of activity in cells overexpressing WT CerS5. Data are means ± S.D. n = 3. ^a^, *p* < 0.05 compared to WT CerS5; ^b^, *p* < 0.05 compared to CerS5 W22R. The structure of the amino acids is shown, with their side chains in *pink*. *B*, levels of protein expression, ascertained by Western blotting using an anti-HA antibody and anti-tubulin as a loading control, are shown. Molecular weight markers (kDa) are indicated.

We explored the possibility that other aromatic amino acids could substitute for Trp by mutating CerS5^W22^ to Phe, Tyr, and His, which resulted in partial loss of activity (Fig. 3*A*), in contrast to the complete loss of activity upon mutating to the positively-charged Arg. Protein expression and glycosylation remained essentially unchanged, except there was a reduction in levels of the nonglycosylated forms. These results imply that an aromatic residue is essential for CerS activity with the Trp indole ring required for maximal CerS activity.

### An unusually high abundance of Trp in CerS

**Figure 4:**
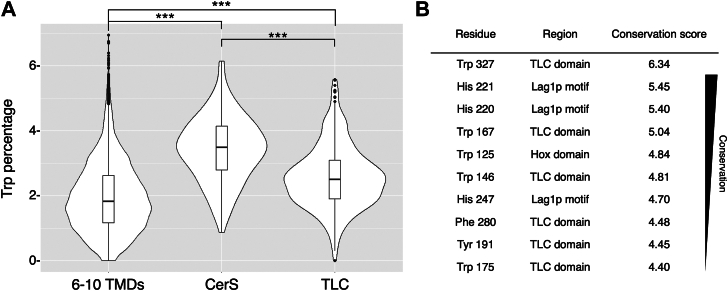
**Trp abundance and conservation in CerSs.***A*, violin plots comparing the percent of Trp residues in proteins with 6 to 10 TMDs, CerSs, and TLC domain-containing proteins (excluding the CerS clade) from the Swiss-Prot database. Sequences with >70% redundancy were eliminated. The boxes within the violin plot represent lower quartile, median, and upper quartile. The whiskers extend to the minimum and maximum values that fall within 1.5 times the interquartile range from the *lower* and *upper* quartiles. Data points beyond this range are considered outliers (represented by *black* dots). The broadness of the violin plot represents the number of proteins at the corresponding Trp percentage level. *B*, the top ten conserved positions of CerSs arranged in descending order according to their Information Content (stack height) values, reflecting conservation score, using the Skylign web server (52). The residue number in the sequence is shown for CerS5. CerS1-6 multiple sequence alignment used for this analysis was obtained from Ref. (21).

To further understand the possible roles of Trp residues in CerS, we examined the abundance of Trp residues in CerS sequences compared to TLC domain-containing proteins (excluding the CerS clade), as well as transmembrane proteins with six to ten TMDs (chosen since this is a similar number of TMDs as found in CerSs). The number of protein sequences varied across each group, with the largest number in proteins with six to ten TMDs (6–10 TMDs, 8613 sequences; CerSs, 161 sequences; TLC domains; 308 sequences). Unexpectedly, the abundance of Trp residues varied significantly between each group (Fig. 4*A*). The average content of Trp residues in proteins containing 6 to 10 TMDs was 1.89 ± 1.05%. In contrast, the CerSs contained a higher percentage of Trp residues (3.35 ± 1.07%), which was also higher than other TLC family members (2.61 ± 0.99%), suggesting a distinct role for Trp in shaping the functional characteristics of CerSs.

Previous studies have shown that the abundance of Trp residues in membrane proteins is higher than in soluble proteins, although the precise reported abundance varies (6, 8, 27, 28, 29). Thus, the percent of Trp residues was reported as 1.2 to 7.1%, although each study used a relatively small subset of proteins (*i.e.* membrane and nonmembrane proteins across eukaryotes from different phyla (8), lysosomal membrane proteins and nonlysosomal membrane proteins (28) and human type 1 single span membrane proteins (29)). The most well-known and well-quoted study suggests that Trp comprises ∼3.1% of the amino acid residues in transmembrane proteins but only 1.2% in soluble proteins, although this study was based on a small number of bacterial cell membrane proteins (6). We now re-calculate Trp frequency in all nonredundant proteins from the Swiss-Prot database, categorized as (i) all protein sequences (216,303 sequences), (ii) transmembrane proteins (TM) with varying numbers of TMDs (33,668 sequences), (iii) CerS family members (161 sequences), (iv) TLC domain family members (308 sequences), (v) soluble proteins (183,018 sequences) (Table 1, see additional details in Table S1), and (vi) proteins located in the ER (3097 sequences). However, the subcellular location of many of the ER proteins is questionable, with some predicted to be in more than one location; nevertheless, the percent of Trp residues in this family of proteins is 1.70 to 1.74%.

**Table 1:** Amino acid abundance in membrane and soluble proteins from the Swiss-Prot database

Amino acid	All proteins	Transmembrane proteins					CerS∗	Other TLC-domain containing proteins∗	Soluble proteins
		All membrane proteins	1 TMD	2≤TMD≤5	6≤TMD≤10	11≤TMD			
Ala	8.04 ± 3.71	8.09 ± 3.66	7.37 ± 3.75	8.36 ± 3.90	8.36 ± 3.23	9.16 ± 3.10	6.11 ± 1.89	7.40 ± 3.00	8.03 ± 3.72
Cys	1.57 ± 1.95	1.51 ± 1.44	1.68 ± 1.73	1.41 ± 1.38	1.48 ± 1.14	1.27 ± 1.03	1.68 ± 0.84	1.98 ± 0.97	1.59 ± 2.03
Asp	5.29 ± 2.06	3.83 ± 1.83	4.78 ± 1.90	3.54 ± 1.80	3.12 ± 1.32	3.09 ± 1.20	4.51 ± 0.90	3.06 ± 1.00	5.55 ± 1.98
Glu	6.61 ± 2.83	4.64 ± 2.44	6.01 ± 2.67	4.30 ± 2.77	3.53 ± 1.54	3.54 ± 1.38	4.31 ± 1.09	3.11 ± 1.20	6.97 ± 2.74
Phe	3.96 ± 1.96	5.55 ± 2.4	4.33 ± 2.24	6.01 ± 2.69	6.43 ± 2.05	6.35 ± 1.75	7.45 ± 1.48	7.05 ± 1.73	3.67 ± 1.70
Gly	6.84 ± 2.84	6.85 ± 2.72	6.25 ± 2.73	7.01 ± 3.01	7.08 ± 2.43	7.93 ± 1.98	4.57 ± 1.17	5.55 ± 1.70	6.84 ± 2.87
His	2.22 ± 1.34	1.92 ± 1.19	2.08 ± 1.29	1.83 ± 1.26	1.92 ± 1.10	1.63 ± 0.75	2.71 ± 0.80	2.88 ± 0.93	2.28 ± 1.36
Ile	6.06 ± 2.78	7.09 ± 3.15	5.96 ± 2.92	7.59 ± 3.45	7.87 ± 2.91	7.72 ± 2.47	6.98 ± 2.06	6.69 ± 2.54	5.87 ± 2.67
Lys	6.17 ± 3.38	4.73 ± 2.56	5.73 ± 2.94	4.56 ± 2.45	3.95 ± 1.85	3.59 ± 1.50	5.22 ± 1.26	4.24 ± 1.67	6.44 ± 3.44
Leu	9.67 ± 2.95	11.7 ± 3.31	10.2 ± 3.18	12.2 ± 3.44	12.9 ± 2.86	12.7 ± 2.59	11.5 ± 1.49	12.6 ± 2.43	9.29 ± 2.72
Met	2.43 ± 1.24	2.77 ± 1.39	2.41 ± 1.39	2.92 ± 1.52	2.96 ± 1.21	3.12 ± 1.23	2.73 ± 0.77	3.12 ± 1.11	2.37 ± 1.20
Asn	4.16 ± 2.39	3.86 ± 2.16	4.39 ± 2.42	3.73 ± 2.17	3.47 ± 1.81	3.34 ± 1.47	3.15 ± 1.22	3.41 ± 1.40	4.22 ± 2.43
Pro	4.65 ± 2.38	4.52 ± 2.17	5.14 ± 2.70	4.16 ± 2.06	4.20 ± 1.42	4.18 ± 1.17	3.92 ± 1.12	3.80 ± 1.47	4.68 ± 2.41
Gln	3.79 ± 2.08	3.30 ± 1.73	3.96 ± 2.01	3.06 ± 1.68	2.86 ± 1.24	2.74 ± 1.03	2.76 ± 1.02	2.61 ± 1.09	3.88 ± 2.13
Arg	5.55 ± 2.72	4.52 ± 2.10	5.14 ± 2.35	4.41 ± 2.09	4.10 ± 1.70	3.66 ± 1.43	5.07 ± 1.45	4.46 ± 1.47	5.74 ± 2.78
Ser	6.82 ± 2.83	7.36 ± 2.56	7.68 ± 2.93	7.11 ± 2.69	7.16 ± 2.02	7.42 ± 1.78	6.75 ± 1.40	7.34 ± 1.66	6.72 ± 2.86
Thr	5.26 ± 1.95	5.48 ± 1.94	5.61 ± 2.38	5.26 ± 1.84	5.50 ± 1.50	5.57 ± 1.32	5.01 ± 1.30	5.26 ± 1.59	5.22 ± 1.95
Val	6.80 ± 2.36	7.28 ± 2.26	6.74 ± 2.32	7.44 ± 2.46	7.67 ± 1.97	7.78 ± 1.78	6.75 ± 1.54	8.14 ± 1.78	6.71 ± 2.36
Trp	1.10 ± 0.99	1.67 ± 1.15	1.43 ± 1.18	1.73 ± 1.24	1.89 ± 1.05	1.83 ± 0.79	3.35 ± 1.07	2.61 ± 0.99	1.00 ± 0.93
Tyr	2.98 ± 1.61	3.30 ± 1.58	3.07 ± 1.67	3.33 ± 1.69	3.54 ± 1.40	3.38 ± 1.23	5.51 ± 1.25	4.74 ± 1.70	2.92 ± 1.61
Total sequences	216,303	33,668	12,097	9767	8613	3698	161	308	183,018

Data are shown as percent ± S.D. Sequences with >70% redundancy were eliminated.

∗ Indicates sequences from Ref. (21). For further details, see Table S1.

Transmembrane proteins contain a high percent of hydrophobic amino acids such as Leu, Ala, Val, and Ile (comprising ∼34% of the total). In contrast, soluble proteins show a high frequency (∼25%) of hydrophilic residues (*e.g.* Glu, Lys, Arg, and Asp), with Glu the third most frequent amino acid (6.97 ± 2.74%). Cys and Trp are among the least abundant amino acids in both soluble and transmembrane proteins, including the TLC domain-containing family of proteins. Specifically, the abundance of Trp residues in all proteins is 1.10 ± 0.99% and 1.00 ± 0.93% for soluble proteins and higher in TM proteins (1.67 ± 1.15%), although not as high a previously reported. Some variability was observed depending on the number of TMDs. However, the abundance of Trp residues in CerSs (3.35 ± 1.07%) was much higher than in similar proteins (Fig. 4*A* and Table 1). Additionally, Tyr residues [which tend to cluster near the membrane-water interface and stabilize membrane proteins through hydrophobic interactions (30)] are also more abundant in CerSs (5.51 ± 1.25%; *p* < 0.001 between CerSs and other proteins with 6–10 TMDs) than TM proteins with a similar number of TMDs (3.54 ± 1.40%). Interestingly, while Leu and Ala are the most abundant amino acids in both soluble and transmembrane proteins, CerSs are distinguished by a predominance of Leu and Phe. In addition, Gly is less prevalent in CerSs (4.57 ± 1.17%) than both soluble and TM proteins (6.85 ± 2.72% for transmembrane proteins and 6.84 ± 2.87% for soluble proteins; *p* < 0.001 between CerSs and transmembrane/soluble proteins). The higher abundance of aromatic amino acids, including Trp, Tyr, and Phe in CerSs might indicate that these residues play a role in enhancing specific functional properties or structural stability.

### Conserved Trp residues in CerS

To attempt to determine the functional significance of the higher abundance of Trp residues in CerSs compared to other proteins with a similar number of TMDs, we identified the 10 most conserved residues in CerSs [from Ref. (21)], five of which were Trp (Fig. 4*B*). All of the 10 most conserved residues are aromatic amino acids situated within the two major structural domains of CerS, namely in the TLC domain and in the Hox-like domain. The most conserved residue in the CerS family is Trp327 (all numbering refers to human CerS5), followed by His220 and His221. These three amino acids are required for catalytic activity and located in close proximity to the proposed catalytic site where the *N*-acylation of the long chain base might occur (18, 31). Of these ten conserved residues, only one is within the Hox-like domain, namely Trp125, which is positioned at the C-terminal end of the domain, adjacent to the TLC domain in Hox-CerSs. Although it is highly conserved, deletion of Trp125 along with the majority of the Hox-like domain did not impair the catalytic activity in CerS5, suggesting that Trp125 may not be critical for its function (17).

**Figure 5:**
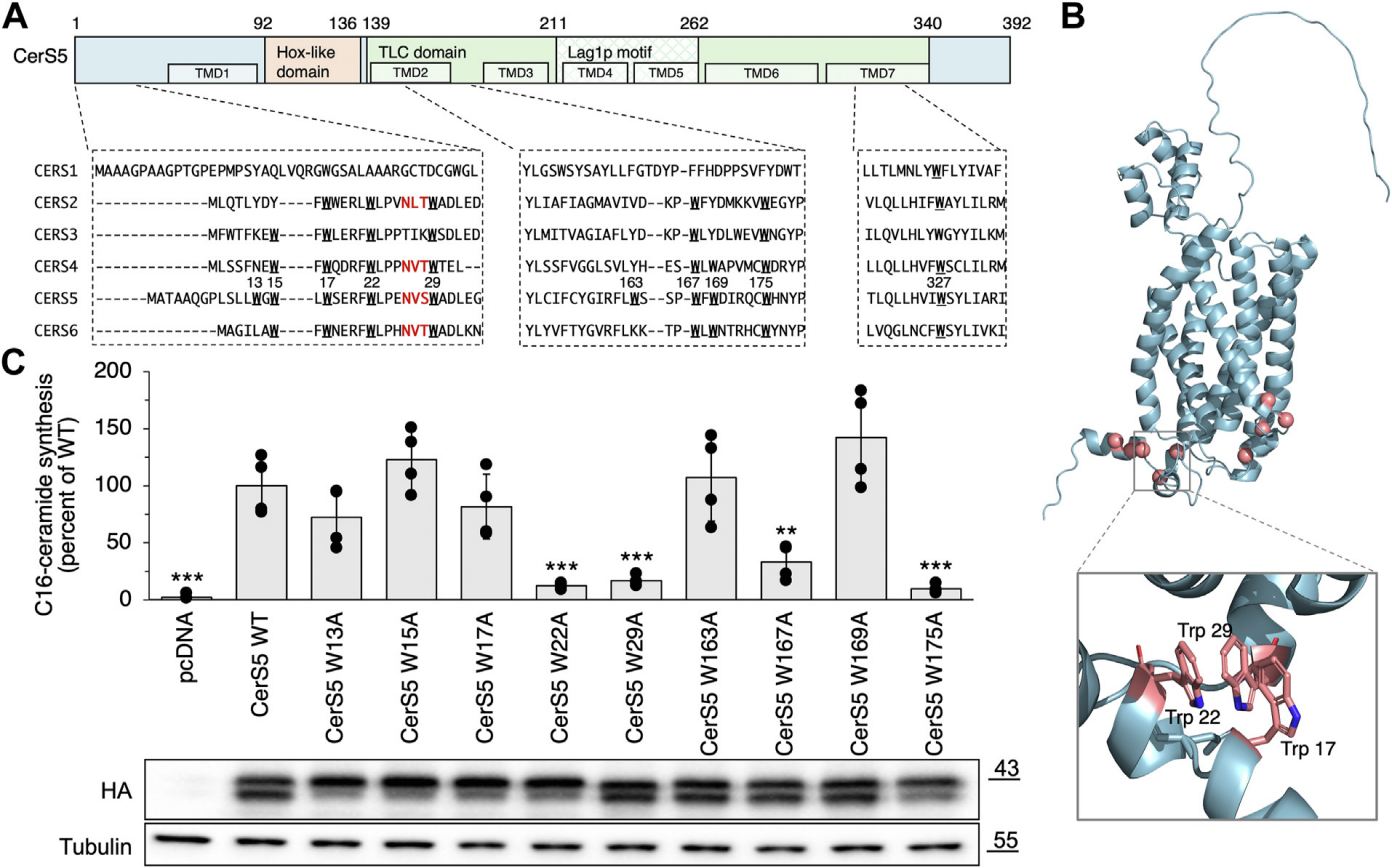
**The effect of mutating conserved Trp residues on CerS activity.***A*, the *upper* panel is a representation of human CerS5 domains with the seven putative TMDs marked according to the AlphaFold 2 model. The *lower* panel shows the multiple sequence alignment of the N-terminus (*left*), the loop between the second and third TMDs (*middle*), and last TMD (*right*) of human CerS. Trp residues in CerS5 are indicated by their amino acid number. The *N*-glycosylation sequence in CerS2 and CerS4-6 is in *red*. *B*, CerS5 model by AlphaFold2 depicted as a cartoon. Mutated Trp residues are indicated as *pink* spheres. The inset shows the specific Trp residues involved in the formation of the aromatic box (40). *C*, homogenates were prepared from WT HEK cells overexpressing the indicated CerS5 mutants. CerS5 activity was assayed using C16-acyl-CoA and 2 μg of protein from a cell homogenate for 5 min. Results are percent activity compared to the WT control ± S.D.; n = 4. ∗, *p* < 0.05. *p*∗∗, *p* < 0.01. ∗∗∗, *p* < 0.001. Levels of protein expression, ascertained by Western blotting using an anti-HA antibody. The glycosylated form is the *upper* band, and the nonglycosylated form the *lower* band. Anti-tubulin was used as loading control. Molecular weight markers (kDa) are indicated. Western blots were repeated at least three times with similar results.

Finally, we explored the relationship between the conserved Trp residues and catalytic activity by mutating most of the conserved Trp residues to Ala at the N-terminus and at the beginning of the TLC domain in CerS5 (between the second and third TMDs) (Fig. 5*A*). All these residues are situated on the ER-lumenal side of the membrane (Fig. 5*B*). Among these residues, Trp167 and Trp175 were identified as highly conserved across eukaryotic CerSs (Fig. 4*B*), while Trp17, Trp22, and Trp29 are broadly conserved within Hox-CerSs (Fig. 2*B*). In contrast, Trp12, Trp15, Trp163, and Trp169 are less conserved. Mutating the conserved Trp residues to Ala decreased CerS activity by >50%, with the exception of Trp17 (Fig. 5*C*). In contrast, mutations in the less conserved Trp residues (Trp13, Trp15, Trp163, and Trp169) maintained activity levels comparable to WT CerS5. Interestingly, mutations of Trp residues positioned before the *N*-glycosylation motif NXS (Trp13, Trp15, Trp17, Trp22) resulted in less expression of the nonglycosylated form (Fig. 5*C*), regardless of the effect on activity.

## Discussion

Using phylogenetic analysis, site-directed mutagenesis (14, 17, 32), and *in silico* analysis (31), much progress has been made in understanding CerS structure and function over the past 20 years (33). Among these are findings that certain mutations are associated with human diseases, such as the CerS3^W15R^ mutation, which causes congenital ichthyosis (19). This discovery motivated the observations generated herein where we show that the N-terminus of Hox-CerS is enriched in conserved Trp residues. As a follow-up, we also demonstrate that Trp abundance in the CerS family is significantly higher than that found in all TM proteins in the Swiss-Prot database that have a similar number of TMDs and that the Trp residues are found in the TMDs near the ER lumen and in a cluster in the N-terminal lumenal region.

Past studies, specifically by von Heijne *et al.* (6), indicated that Trp is approximately ∼3-fold more abundant in membrane proteins than in soluble proteins. Our current analysis of all proteins in the Swiss-Prot database differs from this earlier study inasmuch as we find an ∼1.7-fold difference in Trp abundance. This discrepancy might be attributed to the fact that the von Heijne study was based on a limited dataset of bacterial inner membrane proteins, comprising only 20 membrane proteins and 72 cytoplasmic proteins, in contrast to our study which used a more recent and thus more comprehensive dataset of annotated proteins spanning a wide range of species across all domains of life.

Having shown a lower enrichment of Trp residues than previously described, we were nevertheless surprised to observe that Trp abundance in the CerSs (and to a lesser extent in the non-Hox CerSs) is significantly higher than in other similar multispanning membrane proteins. Moreover, Trp abundance in the CerSs was higher than in other members of the TLC domain-containing family. Additionally, five of the ten most-conserved residues in the CerSs are Trp, including the most-conserved amino acid. Trp is one of the least abundant amino acids in proteins, which has been attributed, at least in part, to its energetically expensive biosynthetic pathway (34). Trp is also the most conserved amino acid in the genetic code, displaying minimal propensity for substitution over evolutionary time (35). Trp is the only amino acid (apart from methionine) that is coded by a single codon (UGG), and a single nucleotide substitution in any position of the codon, including the ‘wobble’ position, results in amino acids of very different physicochemical and functional properties [and in two cases, stop codons (36)]. Clearly, mutations in the Trp codon are prone to cause deleterious effects, reinforcing the idea that Trp is selectively localized in specific positions within proteins due to its distinctive properties (37). Consequently, the enrichment of Trp in membrane proteins, including the CerSs, illustrates the importance of this aromatic residue in maintaining protein structure and stability within membranes.

One of the properties of the CerS proteins that was affected by mutation of Trp residues was *N*-glycosylation (38). In CerS2 and CerS4-6, a conserved N-glycosylation motif is present, with CerS5 the only CerS containing an NXS sequon, while the others contain an NXT sequon. N-glycosylation occurs less frequently at NXS compared to NXT (39), which is consistent with our findings, as CerS5 is generally partially glycosylated (14). Mutations replacing the Trp residues with Ala before the *N*-glycosylation site in CerS5 led to an alteration of the glycosylation pattern, implying that Trp might inhibit, or affect N-glycosylation, potentially *via* steric hindrance of the preceding positions.

Aromatic amino acids such as Trp exhibit a nonrandom distribution at interfaces, often favoring the lipid–water interface (29). In the Hox-CerSs, Trp residues are localized near the membrane interface, forming clusters in close proximity to the lipid–water interface of the ER membrane lumenal leaflet. This asymmetry may be related to the asymmetric distribution of lipids between the two leaflets of the lipid bilayer, since certain lipids may provide more favorable interactions with Trp side chains (27). In contrast, TMDs of integral membrane proteins in post-ER organelles exhibit a different asymmetry inasmuch as Trp residues are located in the region of the TMDs at the outer leaflet of the Golgi apparatus (4). Whether this is due to the different lipid composition of the ER compared to the Golgi apparatus, or whether this further highlights the unique distribution of Trp in the CerSs, is not known.

Examination of the cryo-EM structure of human CerS6 (40) suggests the existence of an ‘aromatic box’ involving specific Trp residues at the N-terminus together with phosphatidylcholine, one of the major lipid constituents of the ER membrane (41), whereby the side chains of conserved Trp9 and Trp21 nestle the quaternary nitrogen of the phosphatidylcholine *via* cation-π interactions, while Trp14, although not directly in contact with the choline head group, likely supports the structural formation of the lipid-binding site (40). A similar arrangement is predicted by AlphaFold2 for CerS5 where Trp17, Trp22, and Trp29 (corresponding to Trp9, Trp14, and Trp21 in CerS6) are positioned in close proximity. Single point mutations of these residues in CerS5 to Ala resulted in a significant loss of enzyme activity, with the exception of Trp17 which retained activity. This suggests that Trp22 and Trp29 are critical for maintaining CerS function, potentially through their role in stabilizing interactions with membrane lipids. However, the strength of these interactions is not the same for all lipids; for instance, an ethanolamine head group is more likely to interact *via* cation-π interactions with Trp compared to choline. This difference is caused by steric hindrance due to the bulkier choline group (42). Together, many of these structural details are consistent with our recent proposal that lipid bilayers are ‘finely-tuned molecular assemblies’ such that the atomic interactions between membrane proteins and lipids are fine-tuned to an unexpectedly precise extent (43).

Finally, we note an unexpected difference in the distribution of Trp residues between Hox-CerSs and non-Hox-CerSs, particularly at the N-terminus. This finding is intriguing given that the Hox-like domain and the N-terminus are not in direct contact in the predicted structures of the CerSs and are not known to interact. At present, there is no known selective evolutionary pressure that would indicate why these two structural elements should coincide in modern CerS proteins. Also unknown is whether the Hox-like domain and Trp-rich N-terminal cluster co-emerged in the evolutionary history of eukaryotic CerSs. We have previously explored questions relating to the emergence and evolution of the SL biosynthetic pathway (44, 45), wherein we highlighted the importance of generating or predicting realistic mutational trajectories of the SL biosynthetic enzymes. The observation of a co-incident Trp-rich cluster with a Hox-like domain may help inform the mutational trajectories associated with the CerS family. Is there a functional connection between these two structural features? From the present data, it is clear that some Trp residues in the N-terminus of Hox-CerS (Trp22 and Trp29 in CerS5) are critical for enzymatic activity, and we can speculate about possible roles of Trp in CerS structure and function: the organization of Trp at the ER-membrane interface may facilitate π–π interactions with nearby aromatic residues, which may help form oligomers and stabilize the tertiary structure (46). In the context of an oligomer, the Hox-like domain may have a greater contribution to protein stability. Alternatively, the N-terminal Trp residues flanking the N-glycosylation site (another structural feature absent in non-Hox-CerS) may influence the extent of glycosylation and impact protein stability and localization, properties which the Hox-like domain could also affect. The mutational trajectory by which this Trp cluster evolved (47) is currently unknown, as its relationship to the evolution of the Hox-like domain but the remarkable nature of the finely-tuned interactions between a membrane protein such as the CerSs, the glycosylation machinery, and the lipid bilayer is worthy of note.

## Experimental procedures

### Materials

NBD-Sphinganine (NBD-Sph) and fatty acyl-CoAs were from Avanti Polar Lipids. Defatted-bovine serum albumin, a protease inhibitor cocktail, anti-hemagglutinin (HA), anti-tubulin antibodies, and PEI were from Sigma-Aldrich. An enhanced chemiluminescent detection system and a BCA reagent kit were from Cyanagen. Silica gel 60 TLC plates were from Merck. All solvents were of analytical grade and were purchased from Bio-Lab.

### Bioinformatics

Alignments were performed with ClustalW 2.1 (48). Columns with less than ten sequences were removed from the alignment for subsequent analyses. Logos were created with WebLogo3 (49) using the Chemistry amino acid coloring. The Swiss-Prot protein database, Release 2021_03 (canonical isoforms only) was downloaded on November 3, 2021 (50). A table with annotation was downloaded on the same day, with the field ‘Transmembrane’ taken as a column. The number of transmembrane domains was calculated from this column. Nonredundancy was performed with CD-HIT version V4.8.1 using default parameters, except for c = 0.7 (51). This reduced the total number of proteins in the analysis from 565,254 to 216,303. Nonredundancy was performed on each transmembrane subgroup separately. Conservation score was calculated from the multiple sequence alignment of the CerS clade (21) using the Skylign web-server (52). Amino acid composition was calculated using the nonredundant datasets (CD-HIT c = 0.7) for all of the data subsets. Composition was calculated for each full dataset using Pepstats in EMBOSS (53). Additionally, the distribution per protein was calculated as follows: the absolute count of each amino acid was calculated, followed by the corresponding percentage composition of each amino acid relative to the total number of amino acids in the given protein. The calculations and plots were generated using R scripts.

### CerS constructs

CerS constructs were subcloned from CerS2, CerS3, CerS4, CerS5, or CerS6 in a pcDNA 3.1(+) vector containing a C-terminal HA tag, using restriction-free cloning. Primers are given in Table 2. All sequences were confirmed prior to use.

**Table 2:** Primers used for cloning

Construct	Primers	
CerS2 W15R-HA	F	CTGGTGGGAACGTCTGCGGCTGCCTGTGAACTTG
	R	CAAGTTCACAGGCAGCCGCAGACGTTCCCACCAG
CerS3 W15R-HA	F	GGAAAGATTCCGGCTTCCTCCAAC
	R	GTTGGAGGAAGCCGGAATCTTTCC
CerS4 W15R-HA	F	GGCAGGACAGGTTCCGGTTACCACCCAATGTC
	R	GACATTGGGTGGTAACCGGAACCTGTCCTGCC
CerS5 W22R-HA	F	GAGCGCTTCCGGCTACCCGAG
	R	CTCGGGTAGCCGGAAGCGCTC
CerS6 W14R-HA	F	CTGGAACGAGAGGTTTCGGCTCCCGCACAATG
	R	CATTGTGCGGGAGCCGAAACCTCTCGTTCCAG
CerS5 W22F-HA	F	GGAGCGAGCGCTTCTTTCTACCCGAGAACGTG
	R	CACGTTCTCGGGTAGAAAGAAGCGCTCGCTCC
CerS5 W22H-HA	F	GGAGCGAGCGCTTCCACCTACCCGAGAACGTG
	R	CACGTTCTCGGGTAGGTGGAAGCGCTCGCTCC
CerS5 W22Y-HA	F	GGAGCGAGCGCTTCTACCTACCCGAGAACGTG
	R	CACGTTCTCGGGTAGGTAGAAGCGCTCGCTCC
CerS5 W13A-HA	F	GGACCCCTAAGCTTGCTGGCGGGCTGGCTGTGGAGCG
	R	CTGCCTCCCATGTGACCATT
CerS5 W15A-HA	F	CTAAGCTTGCTGTGGGGCGCGCTGTGGAGCGAGCGC
	R	CTGCCTCCCATGTGACCATT
CerS5 W17A-HA	F	TGCTGTGGGGCTGGCTGGCGAGCGAGCGCTTCTGGC
	R	CTGCCTCCCATGTGACCATT
CerS5 W22A-HA	F	CTGTGGAGCGAGCGCTTCGCGCTACCCGAGAACGTGAG
	R	CTGCCTCCCATGTGACCATT
CerS5 W29A-HA	F	CTACCCGAGAACGTGAGCGCGGCTGATCTGGAGGGGC
	R	CTGCCTCCCATGTGACCATT
CerS5 W163A-HA	F	CTGCTATGGAATTAGATTTCTCGCGTCGTCACCTTGGTTCTGG
	R	CTGCCTCCCATGTGACCATT
CerS5 W167A-HA	F	GATTTCTCTGGTCGTCACCTGCGTTCTGGGACATCCGACAG
	R	CTGCCTCCCATGTGACCATT
CerS5 W169A-HA	F	CTGGTCGTCACCTTGGTTCGCGGACATCCGACAGTGCTG
	R	CTGCCTCCCATGTGACCATT
CerS5 W175A-HA	F	TGGGACATCCGACAGTGCGCGCATAACTATCCATTTCAGCC
	R	CTGCCTCCCATGTGACCATT

F, forward; R, reverse.

### Cell culture and transfection

WT HEK293T or CRISPR CerS2 KO HEK293T cells [HEKCerS2^−/−^ (14)] were cultured in Dulbecco’s modified Eagle’s medium supplemented with 10% fetal bovine serum, 100 IU/ml penicillin, 100 μg/ml streptomycin, and 110 μg/ml sodium pyruvate. Transfections were performed with the PEI reagent using 8 μg of plasmid per 10 cm culture dish. The cells were removed from culture dishes after 36 to 48 h and washed twice with PBS on ice.

### Ceramide synthase assays

CerS assays were performed essentially as described (54, 55). Cells were homogenized in 20 mM Hepes–KOH, pH 7.2, 25 mM KCl, 250 mM sucrose, and 2 mM MgCl_2_ containing a protease inhibitor cocktail. Protein was determined using the BCA reagent. Cell homogenates were incubated with 15 μM NBD-Sph, 20 μM defatted-bovine serum albumin, and 50 μM fatty acyl CoA in a 20 μl reaction volume at 37 °C (for various times and using various amounts of tissue homogenate as indicated in the figure legends). Reactions were terminated by the addition of chloroform/methanol (1:2, v/v) and lipids extracted (56). Lipids were dried under N_2_, resuspended in chloroform/methanol (9:1, v/v), and separated by thin layer chromatography using chloroform/methanol/2 M NH_4_OH (40:10:1, v/v/v) as developing solvent. NBD-labeled lipids were visualized using an AmershamTyphoonBiomolecular Imager and quantified by ImageQuantTL (GE Healthcare).

### Western blotting

Proteins were separated by 10% SDS-PAGE and transferred to nitrocellulose membranes by Trans Blot Turbo (Bio-Rad). HA-tagged constructs were identified using a rabbit anti-HA antibody (Sigma, 1:10,000) and goat anti-rabbit horseradish peroxidase (Jackson ImmunoResearch, 1:5000) as the secondary antibody. Equal loading was confirmed using a mouse anti-tubulin antibody (Sigma,1:10,000) or mouse anti-proliferating cell nuclear antigen (anti-PCNA) antibody (Santa Cruz, 1:500) and goat anti-mouse horseradish peroxidase (Jackson ImmunoResearch, 1:5000) as the secondary antibody. Detection was performed using the enhanced chemiluminescent detection system.

### Trp distribution

Predicted CerS structures were downloaded from the AlphaFold2 Protein Structure Database (https://alphafold.ebi.ac.uk/), identified by Uniprot IDs. Of the previously-published 209 CerS clade sequences, five were discarded owing to mismatched sequences between the predicted structure and the updated NCBI records and an additional three were discarded as no predicted structure was available. Finally, ten of the predicted Hox-CerS structures featured the N-terminus placed on the same side of the transmembrane bundle as the Hox-like domain and were therefore rejected, leaving a total of 66 Hox-CerS and 125 non-Hox–containing CerS predicted structures. Membrane bilayer placement was predicted using the Orientations of Proteins in Membranes (PPM3.0) tool (57). The positions of amino acids relative to the predicted membrane bilayer were measured using Zres (https://github.com/tamir-dingjan/zres). Amino acids located within 40 Å of the predicted bilayer boundaries were analyzed. Graphs were produced using Python.

### Statistics

Statistical significance was assessed using an unpaired one-tailed Student’s *t* test for CerS activity assays. Statistical significance of Trp frequency in CerSs was assessed using one-way ANOVA with *post hoc* Tukey HSD test. Statistical significance of Trp distribution in Hox-CerSs and non-Hox-CerSs was assessed using the Mann-Whitney U rank test, as implemented in SciPy (https://www.nature.com/articles/s41592-019-0686-2).

## Data availability

Predicted structural models of the human CerS proteins are available from the AlphaFold Structure Database (https://alphafold.ebi.ac.uk/) under the following UniProt accession codes: CerS1, P27544; CerS2, Q96G23; CerS3, Q8IU89; CerS4, Q9HA82; CerS5, Q8N5B7; CerS6, Q6ZMG9; Tram 1, Q15629. The amino acid frequency dataset was created from the Swiss-Prot protein database, while the list of the proteins containing the TLC domain was obtained from Ref. (21). The authors confirm that the data supporting the findings of this study are available within the article.

## Supporting information

This article contains supporting information.

## Conflict of interest

The authors declare that they have no conflicts of interest with the contents of this article.
